# How proton transfer impacts hachimoji DNA†

**DOI:** 10.1039/d3ra00983a

**Published:** 2023-05-02

**Authors:** Harry Warman, Louie Slocombe, Marco Sacchi

**Affiliations:** a School of Physics and Maths, University of Surrey Guildford GU2 7XH UK; b School of Chemistry and Chemical Engineering, University of Surrey Guildford GU2 7XH UK louie.slocombe@surrey.ac.uk m.sacchi@surrey.ac.uk

## Abstract

Hachimoji DNA is a synthetic nucleic acid extension of DNA, formed by an additional four bases, Z, P, S, and B, that can encode information and sustain Darwinian evolution. In this paper, we aim to look into the properties of hachimoji DNA and investigate the probability of proton transfer between the bases, resulting in base mismatch under replication. First, we present a proton transfer mechanism for hachimoji DNA, analogous to the one presented by Löwdin years prior. Then, we use density functional theory to calculate proton transfer rates, tunnelling factors and the kinetic isotope effect in hachimoji DNA. We determined that the reaction barriers are sufficiently low that proton transfer is likely to occur even at biological temperatures. Furthermore, the rates of proton transfer of hachimoji DNA are much faster than in Watson–Crick DNA due to the barrier for Z–P and S–B being 30% lower than in G–C and A–T. Suggesting that proton transfer occurs more frequently in hachimoji DNA than canonical DNA, potentially leading to a higher mutation rate.

## Abstract 2

Hachimoji DNA is a synthetic nucleic acid extension of DNA formed by an additional four bases that can encode information. We examine the possibility of proton transfer between the hachimoji bases, which can result in base mismatch under replication.

## Graphical abstract

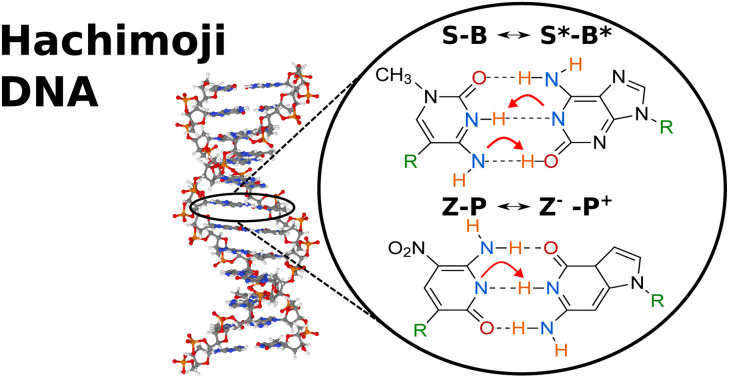

## 1 Introduction

DNA is a crucial part of life, allowing the construction of cells and proteins. DNA stores vast amounts of information in sequences of specific molecules known as bases. The structure of the DNA was proposed by Watson and Crick in 1953,^1^ which was inspired by the conditions proposed by Schrödinger,^2^ which state that the structure should be able to store information and undergo Darwinian evolution.^2,3^ In Watson and Crick's original paper, it was proposed that the four bases consist of four molecules adenine (A), thymine (T), cytosine (C), and guanine (G).^3,4^ These bases can then pair to form the famous double helix structure. In the Watson and Crick model (WC), the base pair must be a purine–pyrimidine pair where A and G are purine molecules, and T and C are pyrimidine molecules.^4,5^

The WC model is of fundamental importance in biology as it shows how DNA carries the genetic information that supports all life on Earth. However, the WC model does not answer why only four DNA bases exist and why those specific molecules are used as the DNA bases.^4^ Work by Szathmáry suggests, through his mathematical model, that the four DNA bases could be one of the most optimal encoding set able to sustain life due to the DNA polymerase's ability to maintain fidelity^3,6^ as more pairs mean more matches polymerase has to account for. While Schrödinger's conditions state that the structure must be a particular form of a purine–pyrimidine pair,^2,5^ developments in synthetic biology have shown that WC bases are not the only potential molecules that could sustain life,^7^ and that evolution could have proceeded with a diverse set of alternative bases.

In 1990 Piccirilli *et al.*^7^ proposed a new set of DNA based on the original WC bases and suggested two additional bases that could potentially sustain life.^2,7^ The work inspired the first generation of artificially expanded genetic information systems (AEGIS).^8,9^ Over the last few years, the number and nature of the bases included in AEGIS have been refined to improve their stability and expand the genetic alphabet from 4 to 12. However, most of the AEGIS research ignores the viability of synthetic bases to be used in nature to sustain life.^9^ In 2020, Hoshika *et al.* determined the viability of the bases included in AEGIS and proposed another type of synthetic DNA, known as hachimoji DNA,^10^ based on a novel set of bases. The difference between hachimoji and AEGIS DNA is that hachimoji DNA consists of only 8 bases instead of the 12 present in AEGIS. The 8 bases selected from AEGIS by Hoshika *et al.* could, in principle, sustain life because they fulfil the structural and thermodynamic requirements proposed by Schrödinger.^2,10^ Hoshika *et al.* verified the structural requirement by attaching hachimoji DNA to a leukaemia virus and seeing if the structure behaves similarly to canonical DNA.^10^ In addition, the thermodynamic requirements were tested by measuring physicochemical properties such as the Gibbs free energy and melting temperature of hachimoji DNA.^10^

**Fig. 1:**
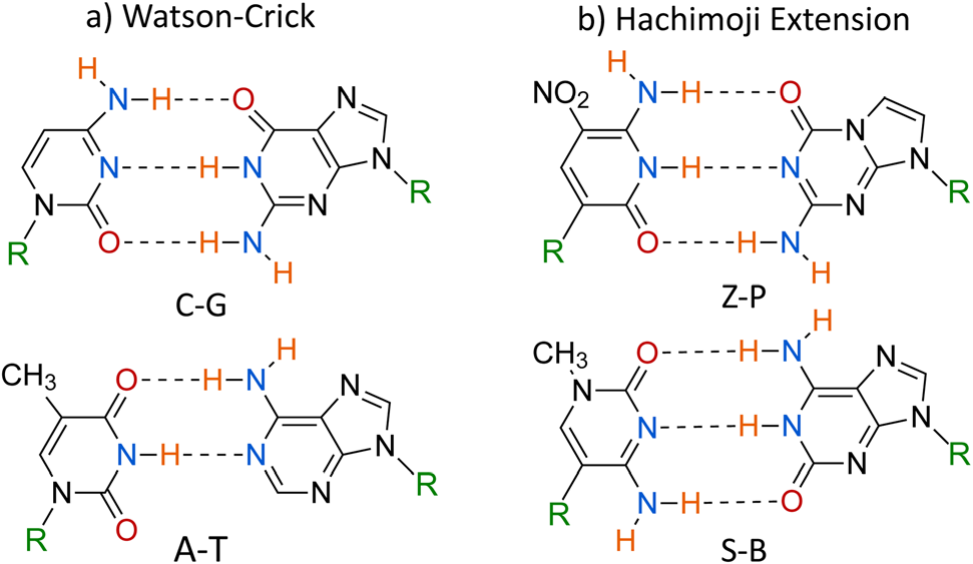
A scheme that shows all base pairs proposed in hachimoji DNA by Hoshika.^10^ (a) corresponds to the standard Watson–Crick pairs, while (b) corresponds to the analogous hachimoji extension. The letters represent the atoms in the bases, while R is where the base connects to the phosphate backbone.

The 8 bases forming hachimoji DNA are shown in Fig. 1. The extra DNA bases increase the information density of the DNA as more pairs can be formed. While hachimoji bases do not occur in nature, these bases could potentially be observed on exoplanets and constitute the genetic material of alternative life forms.^7,10^ Furthermore, it has been proposed that these bases could be used in medicine to treat diseases such as HIV and hepatitis C.^7,10^ Due to hachimoji DNA behaving similarly to Watson–Crick DNA in a leukaemia virus and mostly matching the expected thermodynamic properties, Hoshika *et al.* concluded that hachimoji DNA could be viable in life as it meets Schrödinger's requirements.^2,10^

In this paper, we investigate the resilience of hachimoji DNA to spontaneous mutations *via* proton transfer between the water-solvated hydrogen-bonded bases Z–P and S–B shown in Fig. 1. According to Löwdin's hypothesis,^11^ the proton transfer between canonically bonded WC DNA can lead to the formation of the rare tautomeric non-standard form. Consequently, a problem arises when these tautomeric forms become paired in subsequent rounds of replication, as the WC pairing rule breaks down, leading to base pair mismatching and, thus, potential mutation.^11^ Löwdin's hypothesis has been explored in depth by numerous authors.^12–20^ However, recently, computational studies and theoretical models showed that these tautomers can be readily formed *via* quantum tunnelling and could be involved in the DNA replication machinery.^21,22^ For synthetic DNA, one can ask if proton transfer would detrimentally affect its replication fidelity and decrease its applicability as an information storage or in medicine to treat genetic diseases.^7,10^ In addition, the hachimoji DNA system has 8 bases instead of the typical 4, which makes it a unique model for exploring the relationships between DNA structure, stability, and function.

To address these questions, we need to devise a viable route in which proton transfer between the hachimoji bases could produce a set of tautomers, which would eventually lead to a base mismatch after replication, similar to what was suggested by Löwdin for WC base pairs.^11^ We determine the minimum energy pathways connecting the standard S–B and S–P bases to their respective proton transfer products. Finally, the formation rate and relative stability of hachimoji tautomers and zwitterions will be investigated.

## 2 Methods

### 2.1 Density functional theory

Throughout this work, Density Functional Theory (DFT) is employed to determine the electronic structure and properties of the hachimoji base pairs. All calculations were performed with the B3LYP + XDM/6-311++G** level of theory implemented within NWChem.^23–25^ DFT was used due to its efficiency and accurate results.^26–28^ The 6-311++G** basis set was chosen as it provided a similar level of accuracy as more extensive basis sets of similar systems.^13,15,29^ Similarly, the B3LYP exchange-correlation (XC) functional^29–31^ was chosen for its accuracy in describing proton transfer reactions.^14,15^ Dispersion interactions were accounted for with a non-empirical dispersion scheme, the exchange-hole dipole moment (XDM).^23,27^ In this model, accurate dispersion coefficients are obtained without fitting parameters, which allows the calculation of intermolecular and intramolecular dispersion interactions within a single DFT framework. Furthermore, the solvent effects of the surrounding aqueous solution are included by embedding the system in an implicit solvent model (COSMO).^24^ To provide a direct comparison with previous computational and experimental work,^10,32^ we adopt a dielectric constant of 80 to represent the local water solvent. Gheorghiu *et al.*^17^ suggest that B3LYP and XDM result in a satisfactory combination of exchange-correlation functional and dispersion correction to replicate results at much higher accuracy for the DNA environment.

### 2.2 Exploring the reaction path

The hachimoji bases were first constructed using the Python package Atomic Simulation Environment (ASE).^23,33^ Initial structures were optimised with a tolerance of 0.01 eV Å^−1^ using BFGS inbuilt ASE.

To describe the proton transfer mechanism, a potential energy surface (PES) depicting conformational change from the canonical to the proton transfer form needs to be obtained. This work uses a machine-learning approach to the nudged elastic band method (ML-NEB).^34,35^ NEB is an algorithm that finds a path of state with minimal energy that connects the initial and final state together.^34^ The ML-NEB algorithm incorporates a surrogate Gaussian process regression atomistic model to accelerate the convergence rate over the classical NEB approach. We used a force tolerance of 0.01 eV Å^−1^ and a target uncertainty in image energy of 0.05 eV. We use 20 images interpolated along the band-path, providing a sufficiently loose spring constant that does not bias the results; benchmarking tests on the effect of this parameter on the ML-NEB accuracy can be found in ref. 14 and 34. In this work, we adopt the ML-NEB method over NEB, as when it is converged, this method can reproduce an accurate minimum energy pathway within the same accuracy as NEB, but at a fraction of the cost.^34^ However, this is provided that the surrogate ML representation is appropriately converged. A full benchmarking and analysis of errors associated with this method can be found within ref. 34.

To improve the accuracy and remove the uncertainty in the barrier's energy, we run each transition state obtained from the ML-NEB band paths through Sella.^36^ Subsequently, the transition state structures are then further refined. However, the rest of the ML-NEB path is subject to the given uncertainty in energy. Sella is an algorithm used to optimise the molecular structures of saddle points accurately by iteratively diagnosing the hessian matrix for the system to obtain the reaction coordinates as well as refining the forces on each atom.^36^

### 2.3 Classical transfer rate

To evaluate the overall stability of the hachimoji proton transfer products, we determine the over-the-barrier classical contribution to the proton transfer rate using standard transition state theory,^26,37^1
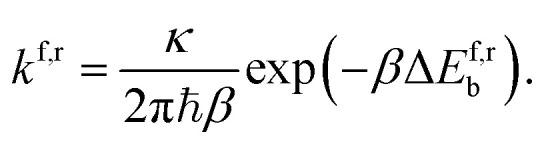
Here *k*^f,r^ denotes the forward, and reverse reaction rates respectively, Δ*E*^f,r^_b_ is the change in energy for the forward and reverse barrier, and *κ* is the tunnelling factor, to link quantum-to-classical contributions. With *β* = 1/(*k*_b_*T*), where *k*_b_ is the Boltzmann constant and *T* the temperature of the system and *ℏ* the reduced Planck constant. The tunnelling factor is a coefficient that accounts for sssquantum tunnelling in the system. There are many methods to obtain this coefficient, and in this paper, we compare two approaches. We adopt the commonly used semi-classical Wentzel–Kramers–Brillouin approximation and compare it to the open quantum system model, which accounts for system-bath interactions with the environment inducing dissipation and decoherence in the quantum system. An approximate expression for the lifetime of a state can be calculated using the inverse of the rate,2
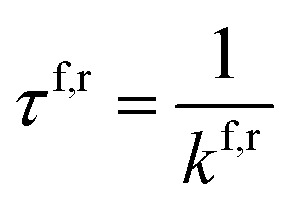


### 2.4 Proton tunnelling using the Wentzel–Kramers–Brillouin approximation

A tunnelling approximation is needed to find the rate at which proton tunnels from one side of the barrier to the other. The Wentzel–Kramers–Brillouin (WKB)^38,39^ method is a semiclassical method commonly used to describe the behaviour of a particle in a potential well. It can be used to approximate the probability of a particle tunnelling through a potential barrier by solving the Schrödinger equation for the system using classical mechanics. This method gives the tunnelling probability as an exponential function of the barrier's width and height and the particle's energy. The WKB method is not exact, but it can provide a good approximate solution in many cases. The WKB method assumes that the wave function decays exponentially through the potential energy barrier. Assuming that at the barrier top, the PES is harmonic, the barrier transmission *P*(*E*) can be obtained using,^38–40^3
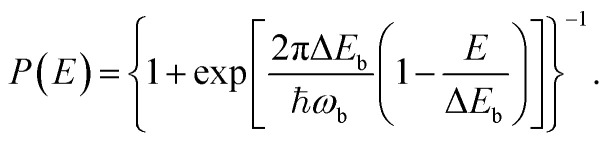
where *ω*_b_ is the imaginary frequency of the barrier (Δ*E*_b_) and is found using^38^4
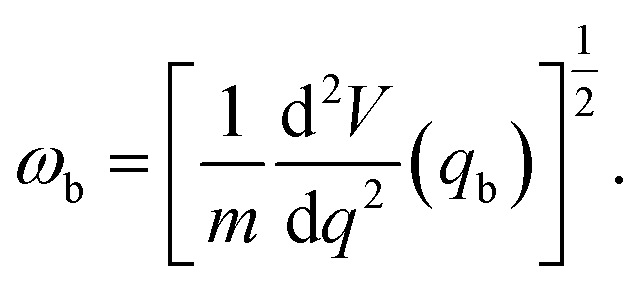
where *m* is the mass of a proton, *q*_b_ is the reaction coordinate (*q*) at the barrier.^38^ Consequently, finding the tunnelling factor using the WKB method results in5
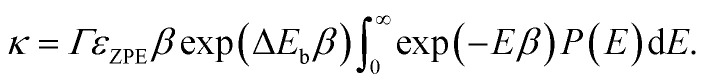
where *Γ* is the transmission coefficient of a proton recrossing the transition state, which we will approximate as 1. *κ* is the tunnelling factor of the system. The *ε*_ZPE_ term corresponds to the ratio between the quantum and classical partition functions for the reactant. This factor thus accounts for the zero-point energy effect in the reactant state,6
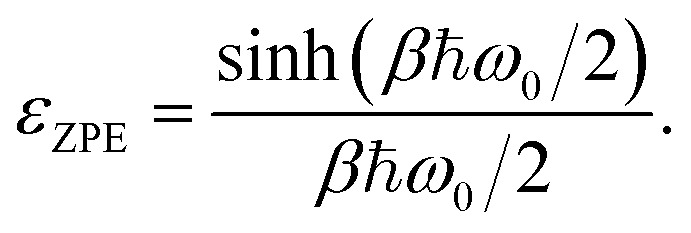
where *ε*_ZPE_ approximated by a harmonic oscillator with frequency *ω*_0_ denotes the spring constant at the bottom of the reactant well, representing the lowest oscillator.

### 2.5 Using an open quantum systems approach

A fully isolated quantum system in biology is unlikely to occur, as the environment constantly interacts with the system. For example, in the DNA environment, there are interactions between the system and the environment through vibrations and collisions with the surrounding solvent and proteins, constantly perturbing the quantum system, which results in dissipation and decoherence. Once a quantum system begins to decohere, we expect classical behaviour to emerge. To describe this transition region, we require a theoretical framework to account for this. The idea of an open quantum system is to incorporate interactions with the local environment in the quantum dynamics. These interactions significantly influence the system's dynamics and result in quantum dissipation, and decoherence, which can either impede or encourage the system's evolution, a phenomenon known as a quantum Zeno or anti-Zeno effect.^41^ Furthermore, the coupling to the environment results in quantum dissipation, such that the information in the system is lost to its environment and decoherence, where a quantum system loses its wave-like properties. The general idea is to couple a system Hamiltonian *Ĥ*_S_ with a bath *Ĥ*_B_*via* an interaction *Ĥ*_I_,

7 *Ĥ*_SB_ = *Ĥ*_S_ + *Ĥ*_B_ + *Ĥ*_I_.

Here the interaction term generates quantum and classical correlations between the system and the environment.^42^ Consequently, we consider a model Hamiltonian for a double-well potential bilinearly coupled to a bath of harmonic oscillators to describe the proton transfer reactions.^22^ The open quantum systems (OQS) approach employed in this study is based on Caldeira and Leggett's quantum Brownian motion model.^43^ In this approach, a proton is embedded in an ohmic bath of quantum oscillators representing the local cellular environment. In the model, we assume that the proton is bilinearly coupled to the bath oscillators and assume that the system's influence on the bath is negligible (Markov approximation). The system bath interactions are integrated over time using the path integral formalism introduced by Feynman and Vernon.^44^ The phase-space formulation of Caldeira–Leggett's model to order 
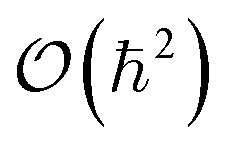
 is written as8
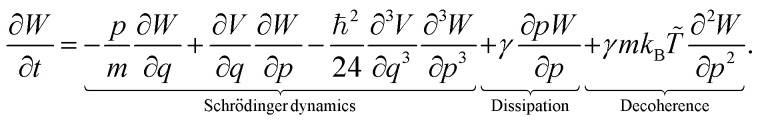
where *W* is the Wigner distribution, a quasi-probability density encapsulating the proton's quantum state as a function of position (*q*) and momentum (*p*) coordinate.^45,46^ Here, *γ* is the phenomenological friction constant that describes the strength of the coupling to the bath^43^ and *T̃* represents the effective bath temperature of the biological environment (300 K). At low temperatures, the effective bath temperature is set to approach the zero-point energy of the system.^43,47–51^ The effective bath temperature is evaluated using:9
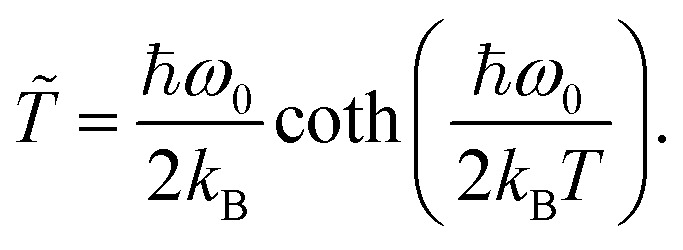
while at high temperature, the equation recovers the standard temperature expression, since *T̃* → *T*. Assuming that the system-to-environment coupling, *γ*, is governed by the fluctuations of the surrounding water molecules, we can postulate that the fastest oscillators in this range to dictate the ohmic spectral density; *γ* = 3900 cm^−1^.^43,52^ Consequently, the oscillations induced by the bath are much faster than the system dynamics; we approach the Smoluchowski limit (*γ* = 3900 cm^−1^ ≫ *ω*_0_). In this limit, the bath induces the separation of timescales between the evolution of position and momentum. Thus, we can then take

10 *P*^QSE^(*q*,*t*) ≡ ∫*W*(*p*,*q*,*t*)d*p*.

In the Smoluchowski limit, the eqn (8) can be rewritten as^53^11
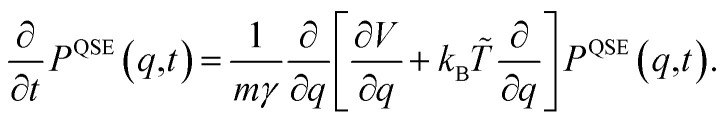
The equation can be solved with the method of lines approach, where the partial derivatives are expanded using a second-order central finite difference with Dirichlet (reflecting) boundary conditions.^54^ Time integration is then performed using Feagin's 14 explicit Runge–Kutta algorithm.^55^

In order to adopt the proton transfer reaction profile into the open quantum systems Hamiltonian, we use a tilted quartic double-well model potential. The double-well potential describes the proton transfer *via* the minimum energy pathway determined from the ML-NEB calculations and assumes that the hydrogen bonding atoms bind the proton at both the donor and acceptor bonding sites. Consequently, creating a double-well or two-minimum potential, which the proton's wavefunction can distribute along. We encode the information obtained from ML-NEB into the height and shape of the barrier and assert that there is a strong repulsion at small distances to donor and acceptor bonding sites, corresponding to steep bounding potential walls at large *q*. Double-well models in the context of tunnelling in a dissipative environment have been used extensively to model proton transfer reactions.^22,38,56–58^ The tilted quartic double-well model we use is given by^38^12
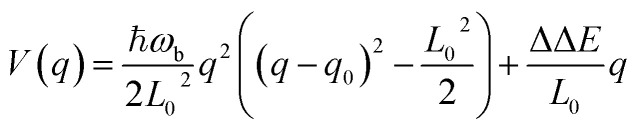
where *q* is the proton position coordinate, *ω*_b_ is the effective spring constant of the barrier, *L*_0_ is the displacement between the potential energy well minima, *q*_0_ a tilt parameter used to control the reaction asymmetry, and ΔΔ*E* is the energy difference between the potential energy well minima. We perform a constrained least-squares fit against the ML-NEB data points to convert to this form. We constrain the fit to capture the reaction asymmetry and barrier values accurately. The final tilted model remains within the ML-NEB uncertainty but also contains steep bounding potential walls at large *q*. Consequently, the model potential is inserted into the open quantum system Hamiltonian, eqn (11). Further information on this transition state searching procedure and its dependence on the parameters are provided in the ESI file.† Furthermore, benchmarking on the accuracy of this approach can be found in ref. 22.

Subsequently, we determine the quantum contribution to the reaction rate by monitoring the flux of the probability passing through the transition state. We initialise the system with a non-stationary distribution thermalised to the reactant well:13
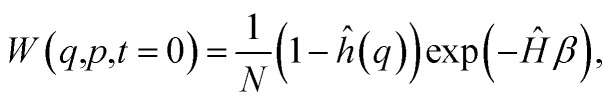
where *Ĥ* = *p*^2^/(2*m*) + *V*(*q*), *N* is a normalisation constant, and *ĥ*(*q*) is a Heaviside step function that projects onto the product side of a transition state dividing surface.

The initial state is propagated forward in time while the proton can be observed tunnelling, and after some characteristic time, the phenomenological rate law can be adopted since the flux of the probability passing through the transition state plateaus and becomes time-independent.^59^ Thus, we determine the rate *via* the time derivative of the probability changes between the left and right-hand well during the plateau.^56,57,60^

### 2.6 Kinetic isotope effect and equilibrium constant

Once the quantum rate has been obtained, we can then calculate the Kinetic Isotope Effect (KIE) to use for further analysis of the mass dependence of the rate. The KIE is simply a ratio between a proton transfer's quantum rate and a deuterated atom's quantum rate. It is given by14
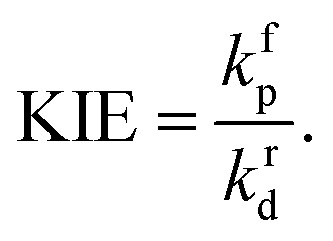
where *k*^f^_p_ is the rate of a proton and *k*^r^_d_ is the rate of the deuterated nucleus.^40^ We use the KIE to probe the reaction mechanism, as it can be a proxy for quantum effects.^61,62^ In our OQS model, we account for both changes in the momentum term in the system hamiltonian and the vibrational changes in the zero-point energy. In comparison, classical mechanisms are primarily independent of mass aside from possible secondary viscosity effects,^63^ whereas quantum is strongly dependent on mass. Consequently, it also provides a probe for experimental approaches.

To investigate the distribution of states at equilibrium, we evaluate the equilibrium constant of the standard and proton transfer products. The equilibrium constant can be found using the ratio of the forward reaction rate and the reverse reaction rate given by,15
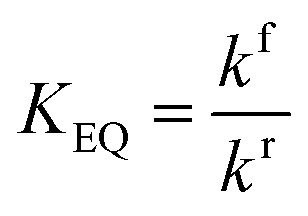
where *k*^f^ is the forward reaction rate and *k*^r^ is the reverse reaction rate.^64^

## 3 Results and discussion

### 3.1 Proton transfer and mutagenic mispairings

In this section, we determine the potential proton transfer pathways where the products lead to a tautomeric or zwitterionic base that could mismatch with a standard canonical base. Here we assume that proton transfer occurs while the bases are hydrogen bonded and are formed before the DNA strands are fully separated by the helicase enzyme responsible for unzipping DNA.

DNA polymerase is an enzyme that catalyses the synthesis of DNA molecules by matching complimentary deoxyribonucleoside triphosphates to the template DNA strand using the standard Watson–Crick base pair rules. Here we assume that the mechanism that causes the polymerase mispairing in WC DNA can be extended to hachimoji DNA. Furthermore, assuming that the proton transfer products could survive the strand separation, we postulate that the polymerase can form a mismatch: tautomer/zwitterionic to a standard base.

**Fig. 2:**
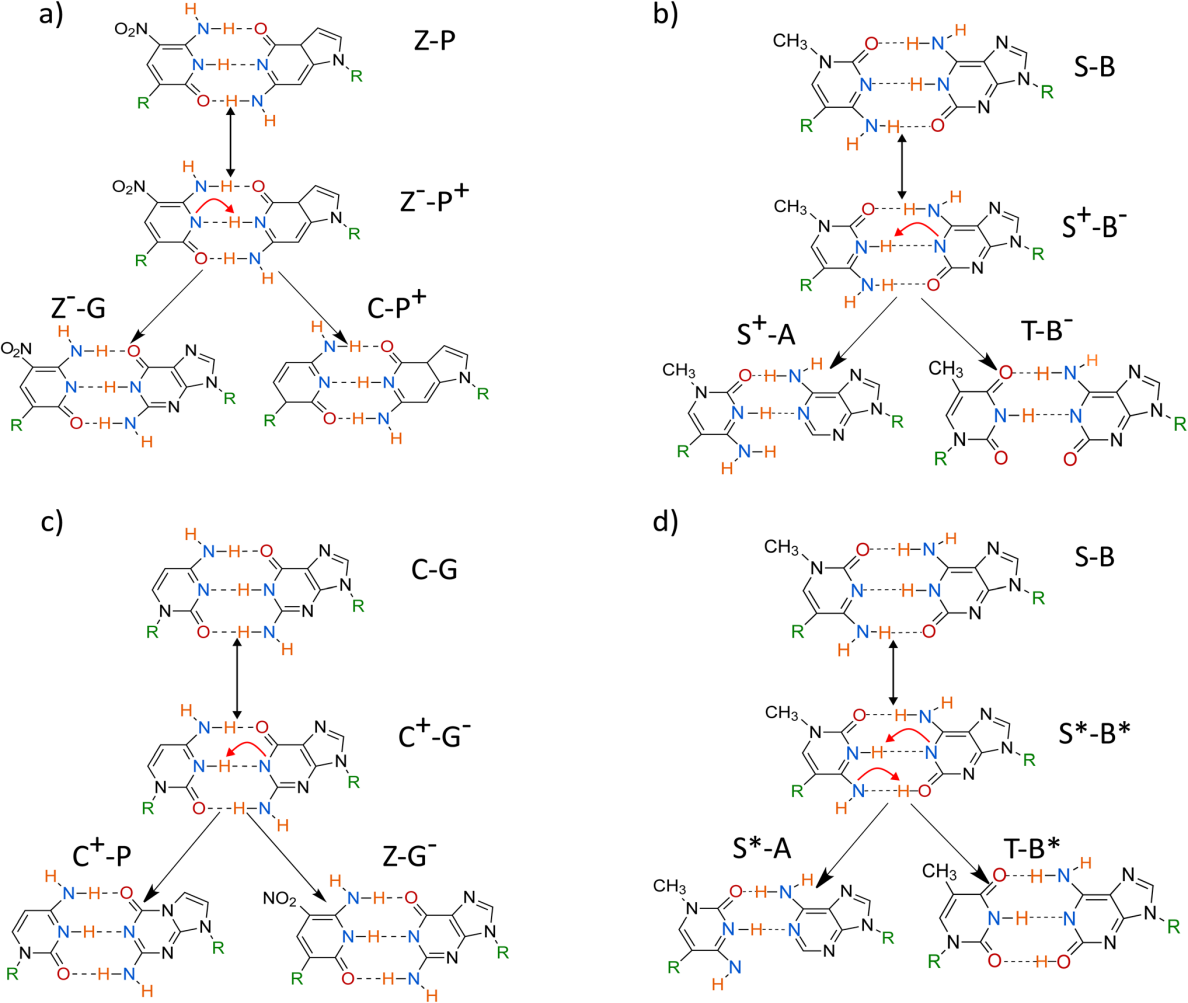
A diagram of the most stable proton transfer schemes that could lead to mutations. Each reaction has three stages; the first stage is the initial proton transfer, forming a zwitterionic (a–c) or tautomeric form (d), which becomes separated under replication. The final stage describes the mismatches with other bases in the polymerase's active site. The ESI† presents a complete picture of all possible pathways, and we present the most thermodynamically stable pairings here.

Consequently, the proton transfer modified hachimoji DNA could evade the polymerase error checking by mimicking the standard, unmodified, Watson–Crick/hachimoji pairing shape. The proposed proton transfer pathways are shown in Fig. 2. Here, the stable pathways are determined by performing DFT calculations on the base pairs in a water solution. If the proton transfer were thermodynamically and kinetically favourable, the replication fidelity would be very low as there would be a large number of base mismatches formed, leading to a very high mutation rate that might not be viable to meet Schrödinger's requirements^2,10^ to sustain life.

To construct the pathways shown in Fig. 2. The initial proton transfer products were chosen based on whether the product would produce a mismatch with another canonical base under subsequent rounds in the replication product. Proton transfer products that can not pair with a canonical base in the hachimoji scheme were ignored due to their biological irrelevance. We have determined ten possible states; the ESI† presents a complete picture of alternative pathways. The geometry of the hachimoji bases was optimised, and the most stable conformers were selected for further study. We performed DFT calculations using these states to determine if these proton transfer states maintained their initial hydrogen bonding patterns. If the protons change their hydrogen bonding during optimisation, be ignored the pathway due to the pairing being thermodynamically unstable and thus not biologically relevant. The only stable proton transfer products and potential mismatches are shown in Fig. 2.

In Fig. 2, the top left panel shows the zwitterionic state of Z–P, where the middle hydrogen transfers along the hydrogen bond. From the zwitterionic product of Z–P, the Z and P base can then mismatch with G and C, respectively. No stable, double proton tautomeric state was found for the Z–P base pair, as the top and bottom protons' most favourable positions are in their standard canonical positions.

The figure also shows both the tautomeric and zwitterionic products for S–B. For the double proton transfer, the tautomeric product is shown in the top right of Fig. 2, and the single proton transfer zwitterionic product is shown in the bottom left of Fig. 2. In the zwitterionic and tautomeric products, the middle hydrogen transfers from B to S. In the tautomeric interaction, the bottom hydrogen is also moved from S to B. The products of these reactions could mismatch in the polymerase, where S and B can mismatch with A and T, respectively.

The C–G base pair was also explored in Fig. 2; the middle proton is moved from the G base to the C base and then mismatches with Z and P, respectively. If one base is zwitterionic and pairs with a canonical base, it would still be stable if the zwitterionic base is switched.

Conversely, Z–P does not present a stable tautomeric form, as the top and bottom proton prefers the canonical positions. After geometry optimisation, we found no reverse barrier for the proton to stay bonded to the alternate base; instead, it reverts to its canonical or zwitterionic form. The zwitterionic form of Z–P presented here has been previously observed in experimental studies.^65^ However, the tautomeric form of Z–P has not been previously observed, which is consistent with our finding. Furthermore, experiments observe the G–Z mispair and suggest that the pairing is highly pH-dependent.^65^

### 3.2 Minimum energy pathways

We determine the minimum energy path connecting the postulated reactions in Fig. 2. Then, using both the initial state and the proton transfer products, we employ the ML-NEB algorithm discussed in Sec. 2.2.

**Fig. 3:**
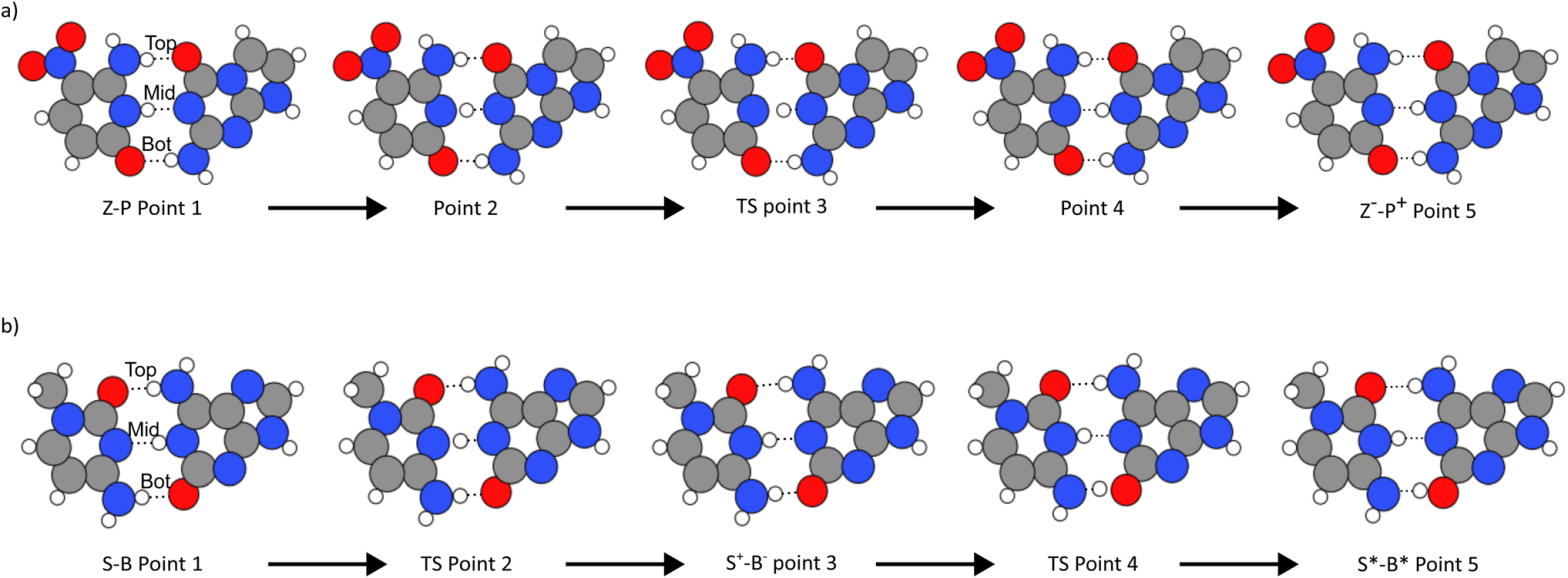
The figure shows the reactions the base pairs undergo to get to the zwitterionic state for Z–P and the tautomeric state for S–B. Where (a) is for Z–P, the middle proton is transferred between the base pairs in a single proton transfer reaction. In (b), S–B undergoes double proton transfer where the bottom and middle protons are exchanged in a step-wise reaction.

Fig. 3 and Table 1 illustrate how the tautomerisation occurs in the hachimoji bases, how the tautomers are formed, and the critical points of the reaction of the Z–P single proton transfer (SPT), shown in panel (a), and the S–B double proton transfer (DPT) shown in panel (b). Fig. 3(a) shows the reaction path of SPT in Z–P, where point 1 is the canonical base pair, and point 5 is the stable DPT product. Point 3 shows the transition state of the reaction. Whereas points 2 and 4 show equidistant points along the reaction path on either side of the barrier, demonstrating the progression of the reaction. The Z–P SPT initially progresses by the stretching of the middle (N–H–N) bond, followed by its transfer from Z to P and the subsequent recoil of the rest of the base. Due to its instability, we could not evaluate a stable DPT product for the Z–P.

**Table 1:** Table showing the change in bond lengths as the proton transfer reaction progresses. The distances between the hydrogens and unpaired atoms in the bases have also been recorded and shown *via* the labels between the bases. The negative sign indicates when the hydrogen has changed which base it is bonded to. Lengths are shown in Angstroms

Reaction	Bond	Point 1	Point 2	Point 3	Point 4	Point 5
Z–P	Top	1.831	1.662	1.669	1.799	1.961
	Middle	1.900	1.592	1.374	−1.749	−1.832
	Bottom	1.888	1.769	1.774	1.736	1.693
S–B	Top	1.806	1.730	1.942	1.996	1.975
	Middle	1.907	−1.456	−1.821	−1.816	−1.892
	Bottom	1.844	1.587	1.626	−1.323	−1.617

In Fig. 3(b), the double proton transfer of S–B is shown. Point 1 is the canonical base pair, and point 5 is the tautomerised tautomeric base pair. Point 3 shows S–B's single proton zwitterionic product, also shown in the bottom left and top right panel of Fig. 2 – as there are two outcomes of the reaction. The S–B double proton transfer reaction is a stepwise reaction with an intermediate zwitterionic product (point 3) in the tautomeric path, where the two protons are exchanged one proton at a time *via* two delimited reaction barriers (points 2 and 4). We determine that the zwitterionic state will form first *via* the movement of the middle hydrogen (N–H–N) of B onto S. Following this, a second transfer pathway where can happen on the bottom bond (N–H–O), where hydrogen transfers from S to B, forming a stable tautomeric DPT product which is neutral in charge. While the mechanism observed here has been unexplored in literature, a decoupled DPT has been observed before, but in WC DNA.^66,67^

**Fig. 4:**
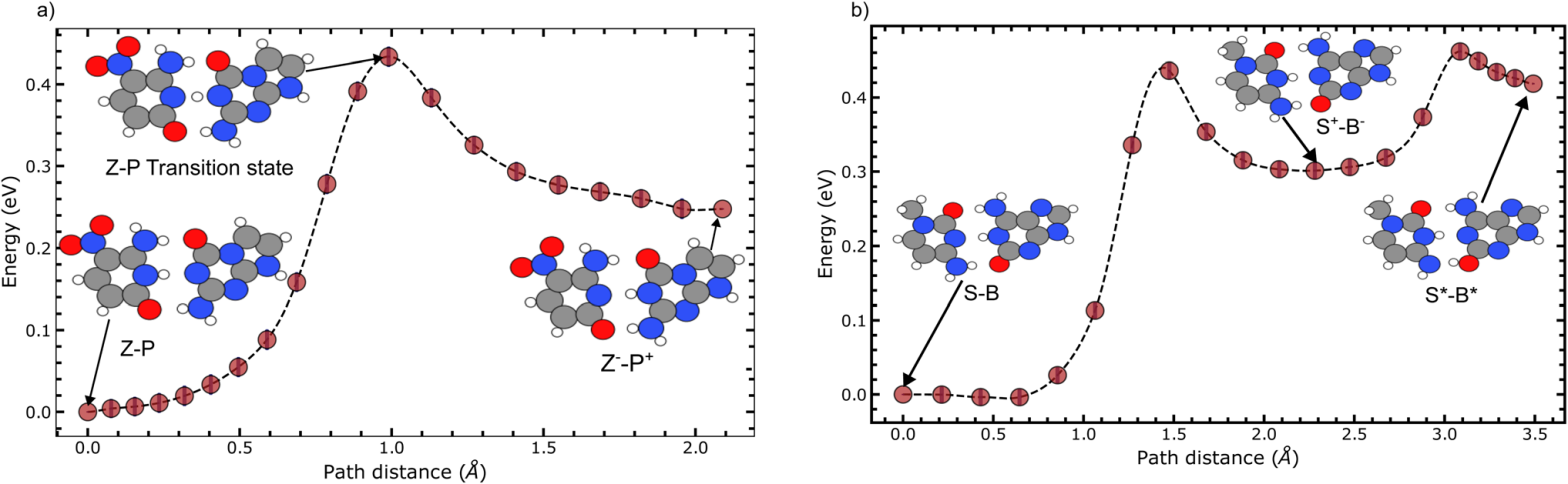
The minimum energy paths for the single and double proton transfer reactions. Here, (a) corresponds to the single proton transfer reaction path for Z–P to its zwitterionic state. Whereas (b) shows the double proton reaction of S–B, where a zwitterionic intermediate state is formed.

In Fig. 4, we evaluate the minimum energy paths for both the SPT of Z–P and the DPT of S–B. Fig. 4(a) corresponds to the zwitterionic reaction of Z–P shown in Fig. 2(a). Whereas, Fig. 4(a) corresponds to the single and double proton transfers in Fig. 4(b) and (d). We do not evaluate the reaction path in Fig. 4(c) as it has already been well documented.^13,14^

For the Z–P reaction, Fig. 4(a), the transition state occurs at 1.05 Å, and the product state occurs at 2.1 Å. Whereas in Fig. 4(b), the intermediary zwitterionic state of S–B occurs at 2.0 Å, while the S–B tautomeric state occurs at 3.2 Å.

The Z–P reaction has a forward reaction barrier of 0.434 eV, which is similar to the first barrier found for S–B (0.413 eV). At the same time, the second S–B barrier is 0.442 eV, which is larger than the first proton transfer barrier. However, as previously mentioned, we could not establish the existence of a second barrier corresponding to double proton transfer in Z–P. Instead, the second proton transfer produces a tautomeric state with a negligible reverse barrier and is thus kinetically unstable. A summary of the energy profiles for these reactions is given in Table 2.

**Table 2:** Table showing properties of the reaction pathways found, the table shows the base pairs forward barrier energy, Δ*E*^f^_b_, the reverse barrier energy, Δ*E*^r^_b_, and the reaction energy (difference between the two barriers), ΔΔ*E*. All the values stated in the table are measured in eV

Reaction	Δ*E*^f^_b_	Δ*E*^r^_b_	ΔΔ*E*
Z–P ⇌ Z^−^–P^+^	0.434	0.186	0.248
S–B ⇌ S^+^–B^−^	0.462	0.135	0.280
S^+^–B^−^ ⇌ S*–B*	0.160	0.043	0.116

Slocombe *et al.* and Gheorghiu *et al.*^13,14^ report energy barriers for single proton transfer in C–G around 0.61 eV.^13^ Comparatively, C–G has a significantly higher (+29%) forward barrier height than the hachimoji bases, with a barrier 0.2 eV larger than the S–B and Z–P reactions. The proton transfer rate is exponentially dependent on the barrier height; this implies that proton transfer is much more likely in hachimoji than in WC DNA. Furthermore, a stepwise and concerted reaction path has been observed for WC DNA.^13,14,21^ The stepwise path contains a similar zwitterionic structure observed in hachimoji DNA and a double proton transfer product. On the other hand, the concerted pathway differs from any reaction paths seen in hachimoji DNA. Gheorghiu *et al.*^13^ report that the local DNA environment strongly influences the dependence on the reaction path type and that the stepwise mechanism is statistically more likely.

The height of the reverse barrier for G–C tautomerisation depends on the employed level of theory^14,15^ and local DNA environment.^13,16–18^ Thus it varies in the literature, with some authors reporting that the proton transfer product state vanishes in some circumstances.^13,15^ The small reverse barrier has often been interpreted as one of the most substantial pieces of evidence for ignoring the role of proton transfer and quantum tunnelling in mutations by implying that the tautomeric state would, in any case, not survive the helicase separation timescale.^15,19,20^ However, recent work suggests that the induced unwinding of DNA by the helicase could simultaneously slow the formation but significantly enhance the stability of tautomeric base pairs and provide a feasible pathway for spontaneous DNA mutations.^21^

For Z–P and S–B, there is a reverse barrier of 0.186 eV and 0.132 eV, respectively, for the zwitterionic reaction. The more considerable reverse barrier indicates that the product is more thermodynamically stable than WC tautomers.

In summary, the lower forward energy barrier means that proton transfer is more likely to occur in hachimoji DNA than in WC and that there would likely be a higher proportion of zwitterionic or tautomeric forms in hachimoji DNA. Provided that these zwitterionic or tautomeric forms can pass through the replication machinery, they could be mismatched, leading to more spontaneous mutations in this DNA scheme. The proton transfer scheme could threaten the ability of hachimoji DNA to be used as genetic information due to the high rate of errors made in replication.

### 3.3 Proton transfer rates: quantum *vs.* classical

This section explores the classical and quantum contributions to the proton transfer rates. We employ transition state theory (described in Sec. 2.3) and use the WKB and OQS approach to account for quantum tunnelling effects. First, we use the energy values obtained from ML-NEB and insert them into eqn (3). Then, we use the PES to obtain the tunnelling rates. The process to obtain the quantum rate can be repeated, but replacing the protons with deuterium nuclei to obtain the KIE of the process. The results of these methods are summarised in Table 3.

**Table 3:** A table summarising the proton transfer rates in the Z–P zwitterionic and S–B tautomeric reactions. Here, *k*^f^ and *k*^r^ is the overall forward and reverse reaction rate, respectively. The rates are measured in s^−1^, and the other metrics are unitless. In addition, *k*_EQ_ denotes the equilibrium constant, and *κ* corresponds to the quantum-to-classical ratio, which gives a metric on how much of the overall rate is comprised of quantum effects. Similarly, KIE measures the sensitivity of the rate on the mass. Finally, *ε*_ZPE_ measures the fraction of the quantum-to-classical partition function accounting for zero-point contributions to the tunnelling

Reaction	Model	*k* ^f^	*k* ^r^	*τ*_f_	*τ*_r_	*κ*	KIE	*ε*_ZPE_	*K*_EQ_
Z–P ⇌ Z^−^–P^+^	WKB	1.31 × 10^7^	1.90 × 10^11^	7.63 × 10^−8^	5.26 × 10^−12^	4.61 × 10^1^	1.96 × 10^1^	2.21	6.91 × 10^−5^
	OQS	4.790 × 10^9^	7.023 × 10^13^	2.09 × 10^−10^	1.42 × 10^−14^	1.497 × 10^4^	1.96 × 10^1^		
S–B ⇌ S^+^–B^−^	WKB	1.27 × 10^7^	6.60 × 10^11^	7.87 × 10^−8^	1.52 × 10^−12^	1.98 × 10^1^	1.22 × 10^1^	1.11	1.92 × 10^−5^
	OQS	1.297 × 10^8^	1.725 × 10^13^	7.71 × 10^−9^	5.8 × 10^−14^	5.114 × 10^2^	1.63 × 10^1^		
S^+^–B^−^ ⇌ S*–B*	WKB	4.47 × 10^10^	4.16 × 10^12^	2.24 × 10^−11^	2.40 × 10^−13^	4.31	4.07	1.07	1.07 × 10^−2^
	OQS	1.005 × 10^11^	9.282 × 10^12^	9.95 × 10^−12^	1.08 × 10^−13^	7.835	3.03		

To determine if the proton transfer products go on to cause a mutation, we must determine first if the transfer is possible. In the last section, we evaluated three viable pathways using DFT; see Fig. 2. However, now we must consider whether the proton transfer products can go on to make a mutation.

Previously, for Watson–Crick DNA over the past several decades, a heated debate has emerged over the biological impact of tautomeric forms.^12,15,19,20^ It is postulated that the proton transfer product must survive the helicase-DNA base opening timescale.^12,20^ Here we assume that this principle also applies to hachimoji DNA. We take the opening timescale to be on the order of 1 ps.^21,68^

From the results found in Table 3, the forward rates show that proton transfer is most likely to occur for the zwitterionic state to the tautomeric state for S–B due to the significant reaction rate, followed by the Z–P reaction and then finally the canonical S–B to the zwitterionic S–B. Contrastingly, the fastest reverse reaction is S*–B* to S^+^–B^−^, followed by S^+^–B^−^ to S–B. In summary, the reverse zwitterionic reaction for Z–P and S–B is more likely to occur than the forward reaction, as indicated by the reaction rates, which should be expected as the reverse barrier is smaller than the forward barrier making it easier to pass energy over or through the barrier. On the other hand, the reverse transfer rate for the second Z–B reaction is two orders of magnitude faster.

We can also use the reaction rates to determine these tautomers' lifetimes, using eqn (2), and then compare them to the lifetimes of WC tautomers found in literature.^14^ The lifetimes found for C–G to C*–G* are 1.17 × 10^−2^ s and 5.49 × 10^−10^ s for the forward and reverse rates. Comparing the forward lifetime shows that the hachimoji reactions are much smaller than those found for Watson–Crick. The lower forward rate suggests that hachimoji DNA is more likely to undergo proton transfer than WC DNA. The reverse lifetimes found for hachimoji DNA are very similar to the lifetimes found in WC as the S*–B* lifetime is on the same order of magnitude as the A*–T* lifetime found, while the lifetime for Z^−^–P^+^ is shorter than the one found for C*–G*, it is most likely due to Z–P being in the zwitterionic state than a tautomeric state rather than a significant difference.

The lifetime of the Z–P product reaction is 1.42 × 10^−14^, whereas, for S–B, it is 5.8 × 10^−14^. Both are much shorter than the helicase opening time of 1 ps.^21,68^ However, recent work^21,68^ highlighted that the proton transfer reaction barrier rapidly increases during the strand separation, suggesting that as long as there is some fast exchange, some product state will likely become trapped by the rapidly rising barrier. Consequently, indicating that a non-trivial fraction of the equilibrium population will be trapped and then be mismatched and cause mutation. Likewise, the second S–B proton transfer product interchanges at a much faster timescale than the helicase opening time. Consequently, there would be a distribution of single and double proton transfer S–B products that are likely to exist.

The WKB quantum tunnelling probability marginally increases the rate of all reactions explored, with the highest tunnelling contribution in the Z–P reaction, which is also signalled by the large KIE value.

However, adopting a more realistic quantum tunnelling model, such as the open quantum system approach,^22,43,59^ accounts for non-trivial system-bath interactions, such as dissipation and decoherence, induced by the local DNA environment. We find that quantum tunnelling significantly increases the proton transfer reaction rate. A quantum-to-classical ratio is now in the 10^1^–10^4^ range. Similarly, the Z–P is the largest affected by quantum tunnelling – reflected in the largest KIE of all the reactions. These results indicate that system-bath couplings, despite causing the system to decohere, lead to significant tunnelling. The WKB method's crudeness can explain the discrepancy between the two tunnelling models. It is known that the WKB method is not sensitive to asymmetrical effects and thus tends to underestimate the rate for proton transfer reactions.^14^

We can use the equilibrium constant to determine how likely each state is to end up in the zwitterionic or tautomeric state. The largest equilibrium constant is caused by the S^+^–B^−^ to S*–B* reaction followed by Z–P to Z^−^–P^+^ and then S–B to S^+^–B^−^. Compared to WC DNA, the equilibrium constants are much larger in terms of orders of magnitude as most reactions in hachimoji DNA are around 10^−5^ while WC DNA's equilibrium constant is around 10^−9^. The higher equilibrium constant demonstrates that proton transfer is more likely to occur in hachimoji DNA than in WC DNA. The small equilibrium constant shown for the S^+^–B^−^ to S*–B* reaction means that the zwitterion state of S–B is favoured over the tautomer state due to the large reaction asymmetry for S*–B* state compared to the zwitterion having quite a deep well. The zwitterion state being favoured is interesting as the system prefers a charged state to a non-charged state.

The KIE can be used to predict which reactions will most likely be affected by tunnelling. Consequently, our calculations provide predictions that could be used by experiments conducted on isotopically substituted artificial DNA. We estimate that the Z–P and the first proton transfer of S–B would have a high isotopic dependence.

**Fig. 5:**
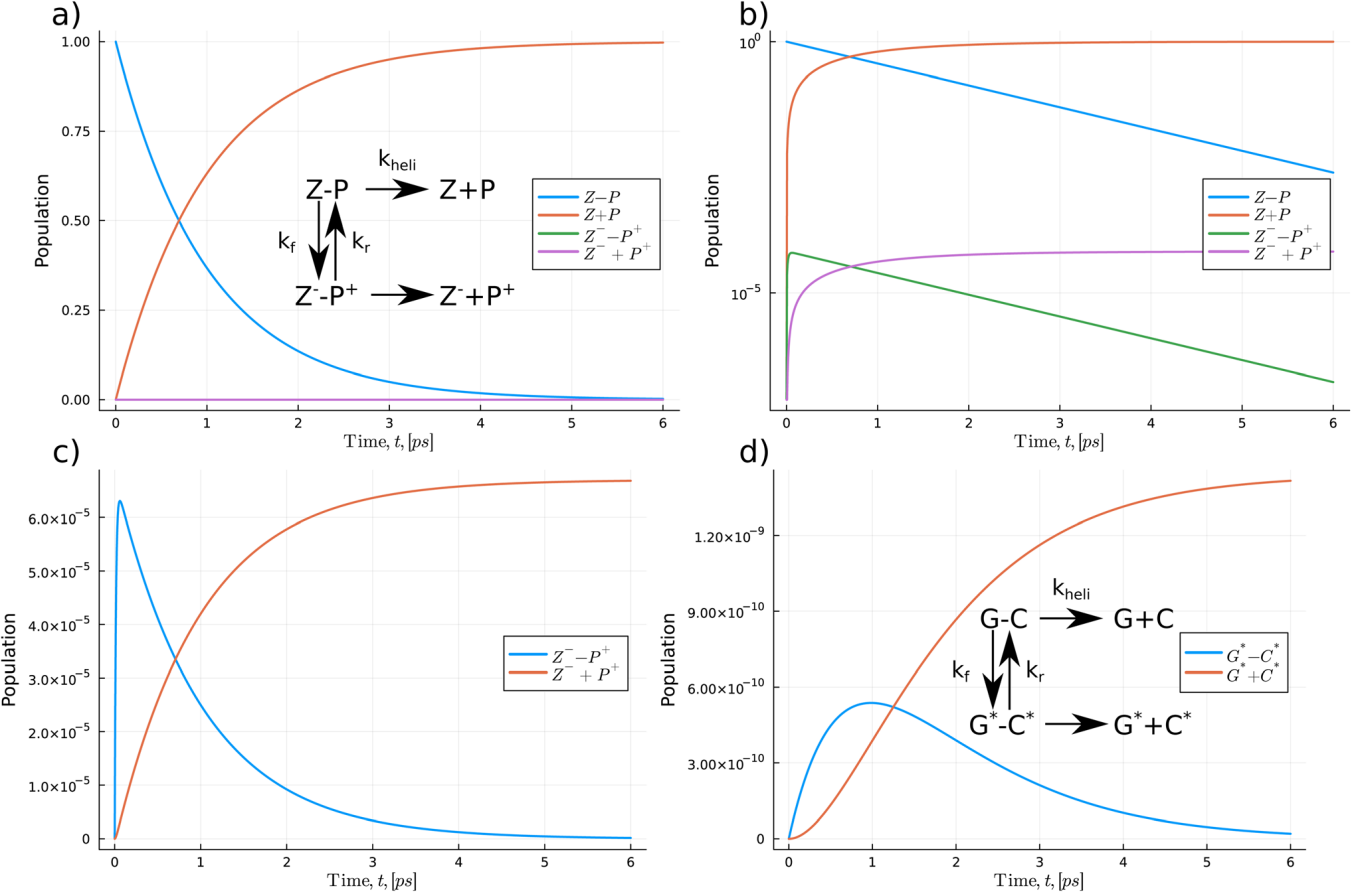
The kinetic reaction network determines the overall population of the proton transfer products for the Z–P reaction. (a) The change in the population of the Z–P base pair, with an insert showing the scheme of the reaction network. (b) Log–linear plot of the populations of all the species in the network. (c) Populations of the Z–P proton transfer products. (d) Comparison to the G–C double proton transfer reaction.

**Fig. 6:**
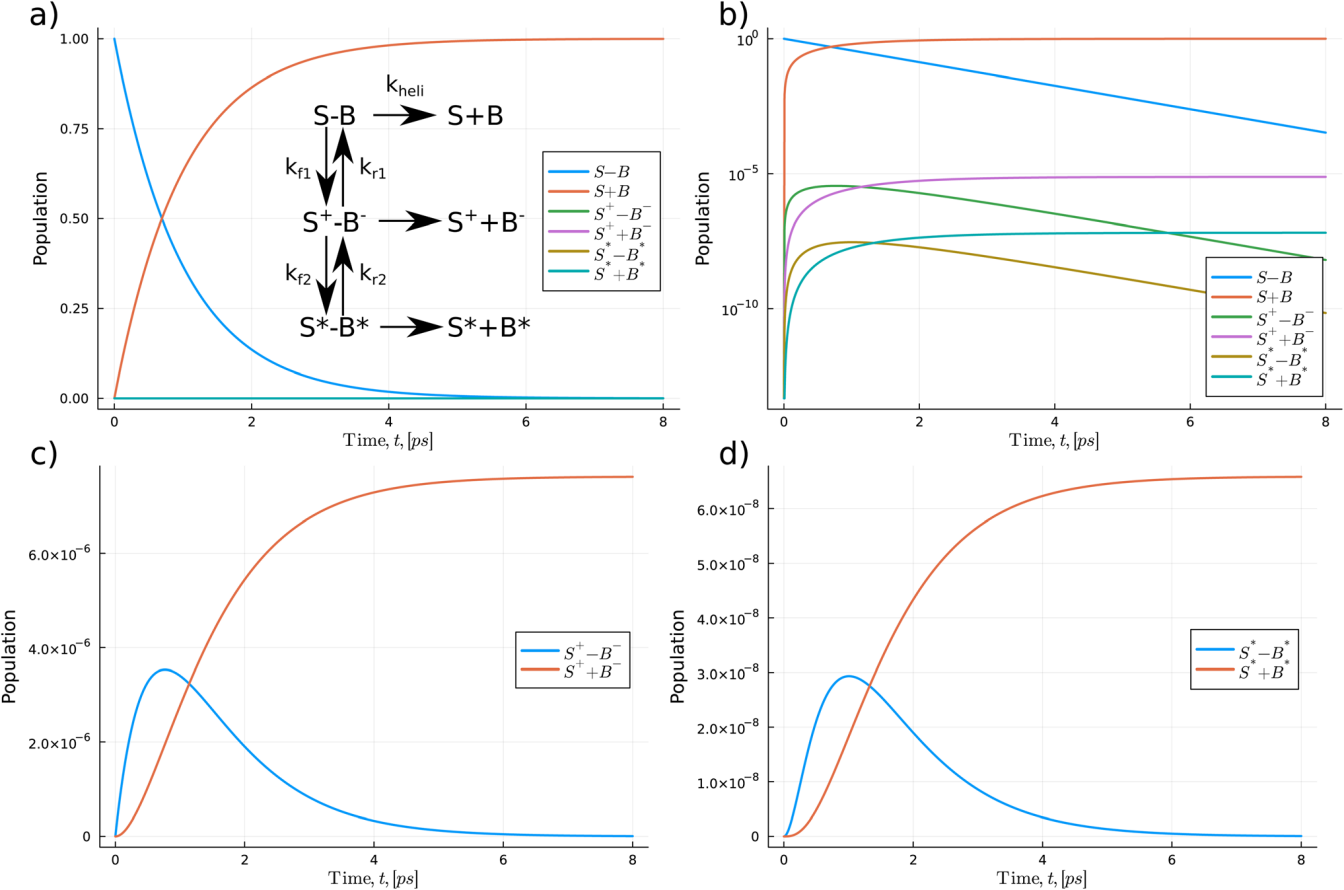
The kinetic reaction network determines the overall population of the proton transfer products for the S–B reaction. (a) The change in the population of the S–B base pair, with an insert showing the scheme of the reaction network. (b) Log–linear plot of the populations of all the species in the network. (c) Populations of the first single S–B proton transfer products. (d) Populations of the second single S–B proton transfer products.

In Fig. 5 and 6, we use a chemical kinetic reaction network approach to determine the population of the proton transfer products that pass through the DNA strand separation procedure, which the helicase enzyme induces. This method describes a series of coupled differential equations where the proton transfer competes with the separation timescale. Consequently, the interaction between the helicase separation time and the proton transfer mechanism acts as a modifier to the equilibrium constant of the proton transfer. To solve the system of the coupled equation, we use the accurate and high-performance Catalyst.jl Julia package.^69^ Here we assume that initially, only the standard forms of the bases are populated. Then as the coupled equations are integrated forward in time, both the proton transfer products and the monomeric forms are populated. We take *k*_heli_, representing the timescale of the helicase separation, to be 1.0 ps.^21^ The authors of ref. 21 used ensemble MD to model the DNA strand separation process. A schematic representation of the reactions is given in Fig. 5 and 6. We use the proton transfer rates using the OQS approach to tunnelling given in Table 3. As previous authors suggested, fewer proton transfer products would exist if the helicase cleaves the hydrogen bonds much more quickly than the proton transfer rate.^15,17,20^ The reaction network approach lets us directly quantify the distributions within the reactants and products.

In Fig. 5(a) initially, the Z–P standard form of the hachimoji base dominates the total population and quickly drops exponentially, reflected by a linear dependence in log space as seen in (b). As time is integrated forward, near the 1.0 ps (one-time constant of the helicase timescale), the separated monomeric forms begin to dominate the overall populations as the strands separate.

From Fig. 5(a) the populations of the proton transfer product, Z^−^–P^+^, and its respective monomeric form appear to have a relatively low population. However, if we replot proton transfer products, see (c), we observe the products taking values on the order of 10^−5^. Initially, the Z^−^–P^+^ dimer population quickly rises to a peak of 6.3 × 10^−5^ before decaying to zero. The separated forms follow a logistic growth trend, increasing to a maximum value of 6.68 × 10^−5^. We also calculate and compare the proton transfer mechanism for G–C and A–T. We take the rate values from DFT calculations on solvated DNA bases,^14^ thus providing a direct comparison for the synthetic case explored here. We assume the reaction network for WC DNA has a similar layout to the Z–P reaction but leads to a double proton transfer. The G–C reaction shows a similar profile has a maximum tautomeric population value of 1.42 × 10^−9^. Overall, hachimoji Z–P proton transfer products have a 10^4^ fold higher population. We also determine the change in populations of the A–T reaction network, and we calculate a final tautomeric population of 1.77 × 10^−10^. Suggesting that A–T proton transfer is 10 times less likely to occur, which agrees with the findings of several other authors.^15,17,20^

In Fig. 6, we explore the S–B pathways. The potential energy surface calculations show that the minimum reaction profile follows a stepwise process for the S–B reaction. Thus, as the reaction profile comprises two decoupled proton transfers, we incorporate this in the kinetic model *via* two separate stages in the reaction; see insert in panel (a). Similar to the Z–P reaction, we find that after 1.0 ps, the monomeric forms of the bases begin to dominate the overall population. However, we see a 100-fold difference between the overall zwitterionic single proton transfer *vs.* double proton transfer products. The maximum zwitterionic products are 7.62 × 10^−6^*vs.* 6.58 × 10^−8^ for the double proton transfer. Thus, indicating that the zwitterionic form of S–B is significantly more likely to occur.

Overall, the maximum population of the proton transfer products for the hachimoji bases saturates at 10^3^–10^5^ times higher than that of Watson–Crick DNA.

## 4 Conclusions

In this work, we report proton transfer mechanisms between the Z–P and S–B base pairs in hachimoji DNA that could lead to the breakdown of the replication pairing rules. We found that single and double proton transfer can occur in S–B, while only single proton transfer can occur in Z–P. We have also shown that the single proton transfer in C–G can mismatch with the canonical bases of hachimoji DNA under DNA strand separation and replication. Furthermore, we determine that A–T can mismatch with the tautomers of S–B. We also compare the proton transfer barrier for synthetic isolated DNA base pairs and isolated Watson–Crick DNA.

We demonstrate that the reaction barriers, 0.434 eV and 0.442 eV, for Z–P and S–B, respectively, are sufficiently low for the proton transfer to occur in a biological setting. Comparatively, for Watson–Crick DNA, the reaction barriers are higher with values of 0.61 eV for C–G and 0.58 eV for A–T.^13,14^ The lower energy barrier suggests that tautomerisation and zwitterionisation are more likely to occur in hachimoji DNA than Watson–Crick DNA, thus leading to more potential mismatches with other bases.

In addition, we calculate the quantum and classical contribution to the proton transfer reaction rate. We applied an open quantum system approach to look into dissipative and decoherence effects induced by coupling to the local DNA environment. We found that the system-bath coupling leads to a 2–3 orders of magnitude increase in tunnelling rates. Due to the fast proton transfer, a significant fraction of proton transfer products could go on to cause replication infidelity. Consequently, the proton transfer mechanism can lead to the breakdown of the steric pairing rules under replication and thus decrease the accuracy of hachimoji DNA information storage. Understanding the intrinsic stability of hachimoji DNA and the relative populations of their zwitterionic and tautomeric forms could impact medicinal chemistry and the development of antisense therapeutics and artificial oligo chemistries.^70–75^

Lastly, we highlight some limitations of the present study and the need for future work to determine the minimum free energy pathway of the proton transfer schemes explored in this paper. Several QM/MM studies on proton transfer in DNA highlight that complex interactions with the solvent, the rest of the DNA structure, and the DNA replisome could significantly alter the DNA shape.^17,76–78^ Furthermore, interactions with the phosphate backbone and stacking interactions with the bases above and below can lead to a sequence dependence on the proton transfer barrier and reaction energy.^16,18,77^ In principle, this could extend to the hachimoji bases and consequently lead to modifications of the proton transfer schemes presented in this work, leading to alterations in the proton transfer barrier and hence tunnelling rates.

## Data availability

The reaction pathways and structures are available on Github.

## Author contributions

L. S. and M. S. conceived and designed this research. H. W. performed the density functional theory calculations. L. S. performed the open quantum systems tunnelling calculations. All the authors contributed to the preparation of the manuscript and have approved the final version of the manuscript.

## Conflicts of interest

There are no conflicts to declare.

## Supplementary Material

RA-013-D3RA00983A-s001
