# Supplementary material for: How proton transfer impacts hachimoji DNA
Source: RSC Adv. 2023 May 2;13(20):13384–96. doi: 10.1039/d3ra00983a (PMC10152326; doi:10.1039/d3ra00983a)
Supplement: RA-013-D3RA00983A-s001 [file RA-013-D3RA00983A-s001.pdf]

Electronic Supplementary Material (ESI) for RSC Advances.
This journal is © The Royal Society of Chemistry 2023

Supporting Information: How Proton Transfer Impacts Hachimoji DNA

Harry Warman,[1,*] Louie Slocombe,[2,†] and Marco Sacchi[2,‡]

[1]*Department of Physics, University of Surrey, Guildford, GU2 7XH, UK.*
[2]*Department of Chemistry, University of Surrey, Guildford, GU2 7XH, UK.*

(Dated: February 13, 2023)

Supporting information to main paper: How Proton Transfer Impacts Hachimoji DNA

CONTENTS

Supplementary Note 1: Possible Tautomers 2

Supplementary Note 2: Reaction Energies 5

Supplementary Note 3: Other Tunnelling Corrections 6

Supplementary Note 4: OQS Tunnelling Using Double-Well Representations 7

* hw00892@surrey.ac.uk
† louie.slocombe@surrey.ac.uk
‡ m.sacchi@surrey.ac.uk

SUPPLEMENTARY NOTE 1: POSSIBLE TAUTOMERS

The proton transfer products were determined to be stable via their hydrogen bonding configuration. However, if the transferred protons returned to their original bonding configuration under geometry optimisation, they are classed as unstable as no significant reverse barrier exists. The proton transfer products were determined by the bases they could mismatch with.

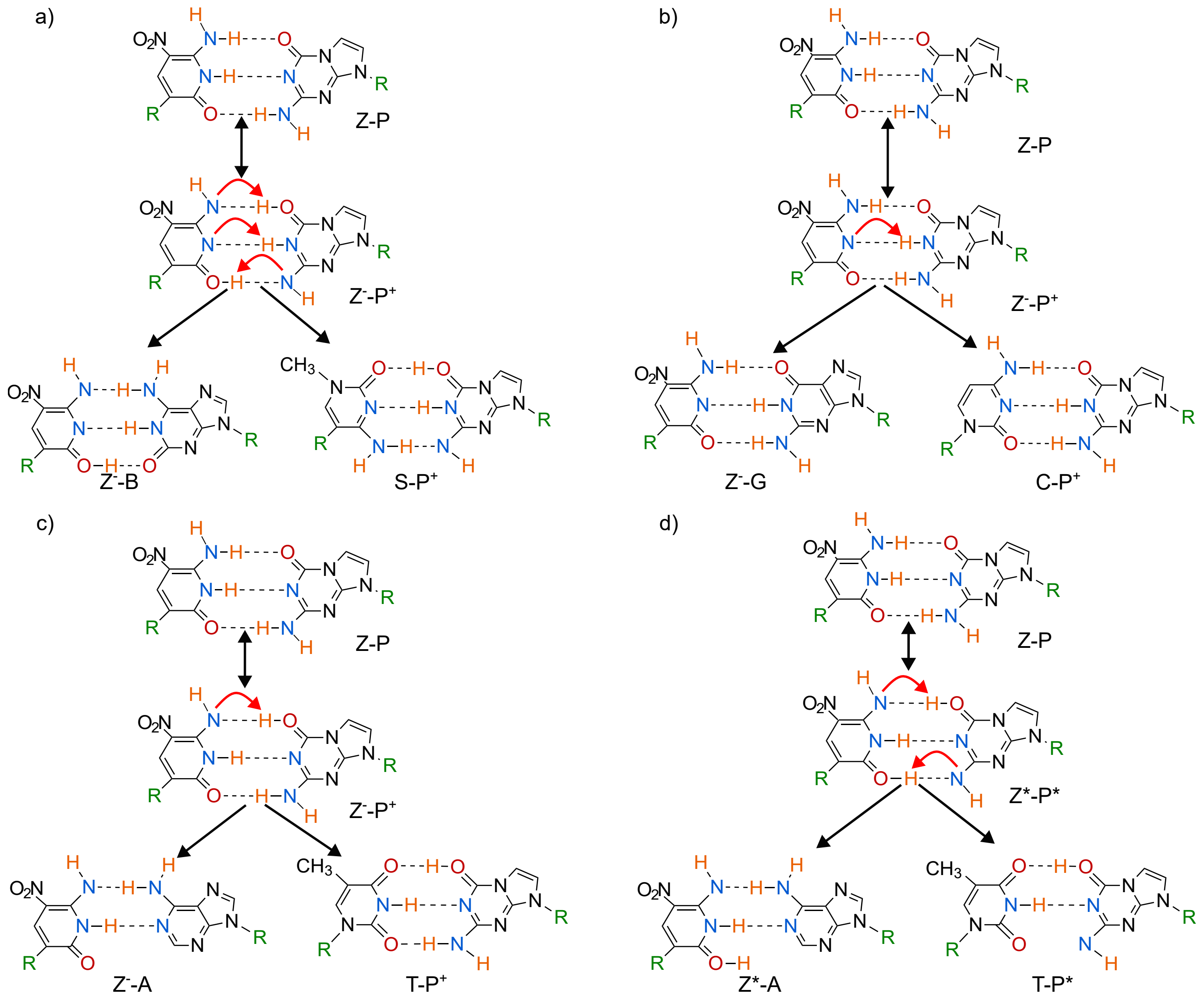

Supplementary Figure 1: Demonstrating all the Z-P reactions tested in the paper and the potential mismatches they can form from proton transfer. Fig 1a) shows the triple proton transfer reaction in Z-P in which a Z-B and S-P can form. Fig 1b) is the single proton transfer in Z-P in which it can mismatch and form Z-G and C-P. Fig 1c) is another single proton transfer of Z-P in which it could mismatch and form Z-A and T-P. Fig 1d) is the double proton transfer reaction in Z-P and shows potential mismatch pairs such as Z-A and T-P. The figure also shows three levels of the reaction, the first showing the canonical base pair, the next showing the proton transfer product and the final showing the system's potential mismatches.

Fig 1 shows all the Z-P proton transfer reactions that were tested in this paper, where fig 1a) shows the triple proton transfer reaction in Z-P, potentially forming mismatch pairs Z-B and S-P. Fig 1b) shows one of the single proton transfers in Z-P where the middle proton is being exchanged from Z to P; this reaction can lead to Z-G and C-P being formed. Fig 1c) shows the other single proton transfer where the top proton is moved from Z to P; this process can create Z-A and T-P. Fig 1d) represents the potential double proton transfer where the top proton moves

from Z to P and the bottom moves from P to Z; the double proton transfer can form Z-A and T-P. From this, it was determined that b) is the only stable product, meaning that the other potential mismatches presented are highly unlikely to be present due to their small lifetime.

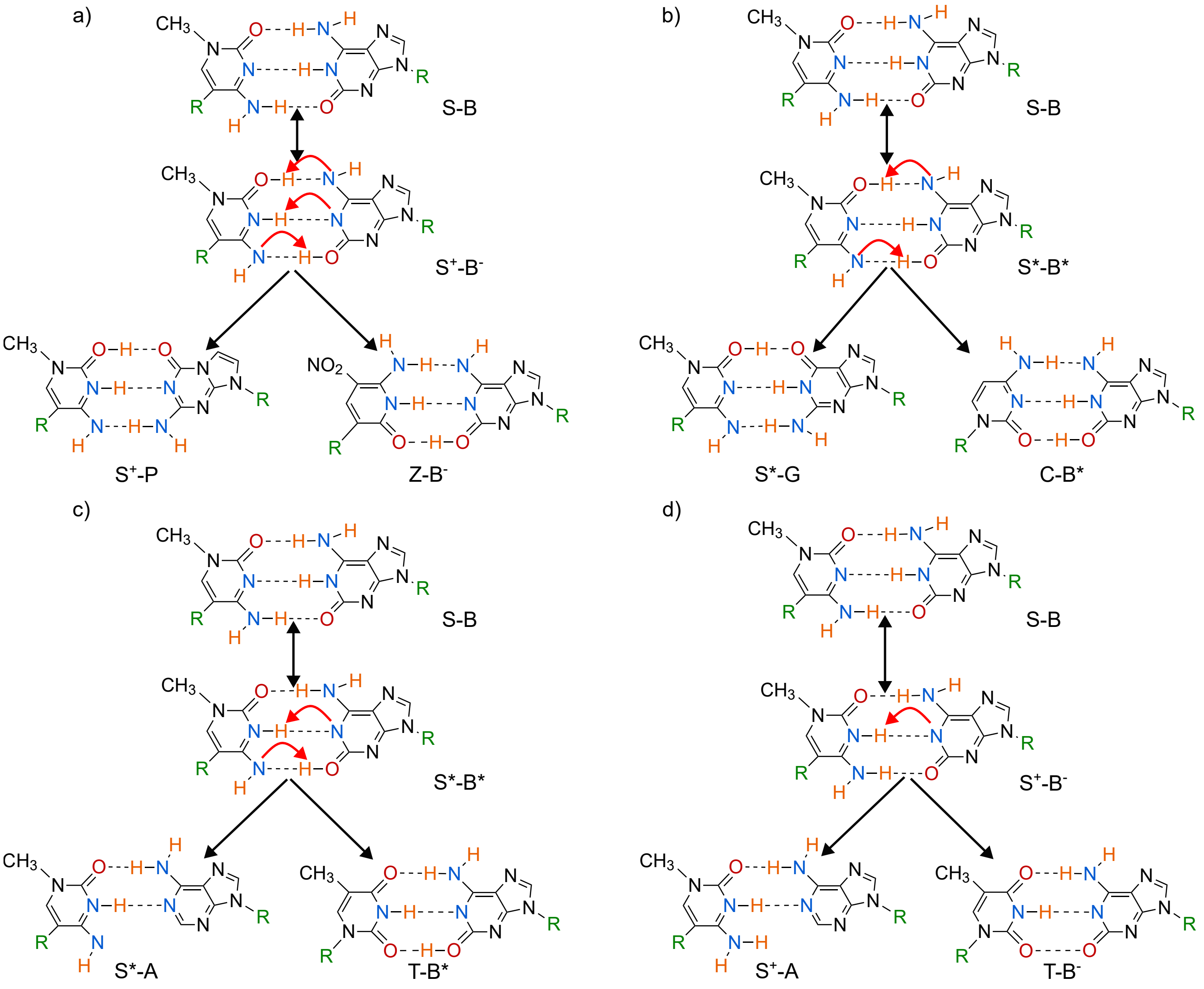

Supplementary Figure 2: Demonstrating all the S-B reactions tested in the paper and the potential mismatches they can form from proton transfer. Fig 2a) shows the triple proton transfer reaction in S-B in which a Z-B and S-P can form. Fig 2b) is the double proton transfer in S-B in which it can mismatch and form S-G and C-B. Fig 2c) is another double proton transfer of S-B in which it could mismatch and form S-A and T-B. Fig 2d) is the single proton transfer reaction in S-B and shows potential mismatch pairs such as S-A and T-B. The figure also shows three levels of the reaction, the first showing the canonical base pair, the next showing the proton transfer product and the final showing the system's potential mismatches.

Fig 2 shows all the S-B proton transfer reactions that were tested in this paper, where fig 2a) shows the triple proton transfer reaction in S-B, which can lead to Z-B and S-P mismatches. Fig 2b) shows one of two double proton transfer reactions that can occur in S-B, where the top proton is being exchanged from B to S and the bottom proton moves from S to B; this reaction can mismatch and form S-G and C-B. Fig 2c) shows the other potential double proton transfer, where the middle proton is moved from B to S, and the bottom proton moves from S to B. This process can create S-A and T-B. Fig 1d) represents the single proton transfer where the middle proton moves from B to S. The single proton transfer can form S-A and T-B. From this testing, it was determined that c) and d) are the only stable product, meaning that the other potential mismatches presented are highly unlikely to be present due to their small lifetime.

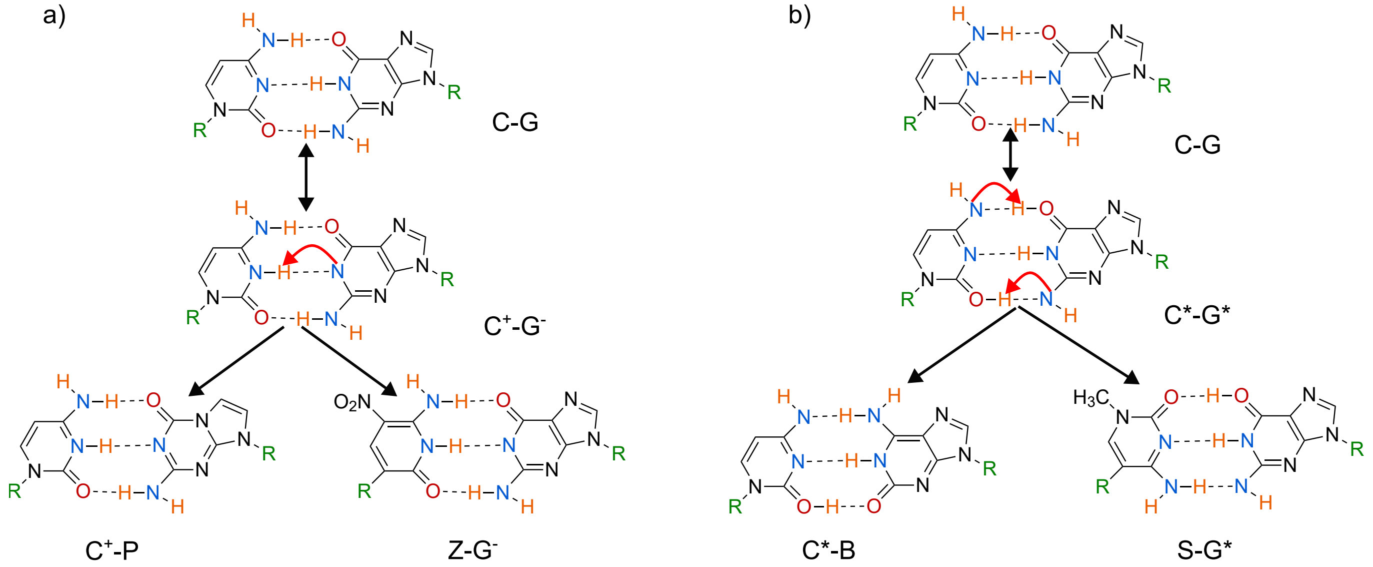

Supplementary Figure 3: Demonstrating all the C-G reactions tested in the paper and the potential mismatches they can form from proton transfer. Fig 1a) shows the single proton transfer reaction in C-G in which a Z-G and C-P can form. Fig 1b) is the double proton transfer in C-G in which it can mismatch and form S-G and C-B. The figure also shows three levels of the reaction, the first showing the canonical base pair, the next showing the proton transfer product and the final showing the system's potential mismatches.

Fig 3 shows how WC DNA can potentially form mismatches with canonical hachimoji DNA bases. Fig 3a) shows the potential single proton transfer that can occur in C-G, in which the middle proton is moved from G to C; this can mismatch and create C-P and Z-G. Fig 3b) shows a potential double proton transfer, where the top proton moves from C to G, and the bottom proton moves from G to C. The double proton transfer in C-G can mismatch and form C-B and S-G. The only stable reaction is a), which agrees with the results found for Z-G.

SUPPLEMENTARY NOTE 2: REACTION ENERGIES

Supplementary Table I: A table that states the base ground state energies for the Z-P, S-B and C-G pairs and their zwitterionic structure and the tautomeric structure for S-B. The energy for the potential mismatches found is also presented. All energies are measured in eV.

Pair	Energy
Z-P	-30652.43
Z^{-}-P^{+}	-30652.19
C-P^{+}	-30640.10
Z^{-}-G	-25534.75
S-B	-26592.68
S^{+}-B^{-}	-26592.36
S*-B*	-26592.25
S^{+}-A	-24556.43
T-B^{-}	-27120.94
S*-A	-24543.67
T-B*	-27133.86
C-G	-25522.70
C^{+}-G^{-}	-25522.39
C^{+}-P	-25534.80
Z-G^{-}	-30639.44

SUPPLEMENTARY NOTE 3: OTHER TUNNELLING CORRECTIONS

A simpler approximation than WKB, known as the Wigner corrections, can find the quantum contributions to the system. Wigner corrections are simple corrections that look at the imaginary frequency at the barrier to approximate the tunnelling factor, given by

$$\kappa = 1 + (\hbar\beta)^2 \frac{\omega_{\mathrm{b}}^2}{24}. \tag{1}$$

While the Wigner corrections are simpler than the WKB approximation, it is less accurate than WKB as it does not consider the shape of the barrier, thus under-predicting the tunnelling factor. Despite this, we can use Wigner corrections in conjunction with WKB to obtain a range of tunnelling factors.

Supplementary Table II: Summarising the proton transfer rates in the Z-P zwitterionic and S-B tautomeric reaction using the Wigner corrections to obtain the tunnelling correction.

Tautomer Pair	k^{f}	k^{r}	κ	KIE
Z-P $\rightleftharpoons$ Z^{+}-P^{-}	1.9267×10^{6}	2.79×10^{10}	6.76	3.08
S-B $\rightleftharpoons$ S^{+}-B^{-}	1.9531×10^{6}	1.0164×10^{11}	3.05	2.18
S^{+}-B^{-} $\rightleftharpoons$ S*-B*	2.6555×10^{10}	2.4753×10^{12}	2.56	2.06

Supplementary Table III: Summary of the potential parameters used to describe the proton transfer reactions. The following parameters are defined by: ω_{b} spring constant of the barrier, L_0 is the displacement, q_0 is the additional tilt parameter, and $\Delta\Delta E$ is the energy difference between the potential energy well minima.

Parameter	Z-P $\rightleftharpoons$ Z^{+}-P^{-}	S-B $\rightleftharpoons$ S^{+}-B^{-}	S^{+}-B^{-} $\rightleftharpoons$ S*-B*
ω_{b}	0.0164 AUT	0.007 57 AUT	0.007 22 AUT
L_0	$2.34\,a_0$	$3.04\,a_0$	$2.11\,a_0$
q_0	$0.315\,a_0$	$-0.160\,a_0$	$-0.185\,a_0$
$\Delta\Delta E$	$0.0121\,E_{\mathrm{h}}$	$0.0065\,E_{\mathrm{h}}$	$0.0018\,E_{\mathrm{h}}$

SUPPLEMENTARY NOTE 4: OQS TUNNELLING USING DOUBLE-WELL REPRESENTATIONS

We describe the proton transfer reaction using model potential in the open quantum systems approach. We describe the proton transfer reactions using a pseudo-one-dimensional reaction coordinate connecting the reactant and product via a transition state barrier. In order to adopt the reaction profile into the open quantum systems Hamiltonian and to determine the reactive flux passing through the barrier, we describe the potential energy surface using a tilted quartic double-well model potential given by the parameters defined in table III.
